# Effects of Sweet-Liking on Body Composition Depend on Age and Lifestyle: A Challenge to the Simple Sweet-Liking—Obesity Hypothesis

**DOI:** 10.3390/nu12092702

**Published:** 2020-09-04

**Authors:** Vasiliki Iatridi, Rhiannon M. Armitage, Martin R. Yeomans, John E. Hayes

**Affiliations:** 1 School of Psychology, University of Sussex, Falmer BN1 9QH, UK; ra294@sussex.ac.uk (R.M.A.); martin@sussex.ac.uk (M.R.Y.); 2 Department of Food Science, College of Agricultural Sciences, The Pennsylvania State University, University Park, PA 16802, USA; jeh40@psu.edu; 3 Sensory Evaluation Center, College of Agricultural Sciences, The Pennsylvania State University, University Park, PA 16802, USA

**Keywords:** sweet taste, gustatory hedonics, body-composition, ingestive behaviour, sugar

## Abstract

Taste hedonics drive food choices, and food choices affect weight maintenance. Despite this, the idea that hyper-palatability of sweet foods is linked to obesity development has been controversial for decades. Here, we investigate whether interpersonal differences in sweet-liking are related to body composition. Healthy adults aged 18–34 years from the UK (*n* = 148) and the US (*n* = 126) completed laboratory-based sensory tests (sucrose taste tests) and anthropometric measures (body mass index; BMI, body fat; fat-free mass; FFM, waist/hips circumferences). Habitual beverage intake and lifestyle and behavioural characteristics were also assessed. Using hierarchical cluster analysis, we classified participants into three phenotypes: sweet liker (SL), sweet disliker (SD), and inverted-U (liking for moderate sweetness). Being a SD was linked to higher body fat among those younger than 21 years old, while in the older group, SLs had the highest BMI and FFM; age groups reflected different levels of exposure to the obesogenic environment. FFM emerged as a better predictor of sweet-liking than BMI and body fat. In the older group, sweetened beverage intake partially explained the phenotype–anthropometry associations. Collectively, our findings implicate underlying energy needs as an explanation for the variation in sweet-liking; the moderating roles of age and obesogenic environment require additional consideration.

## 1. Introduction

Obesity is a global public health concern. According to recent estimates, overweight and obesity affect one in two adults worldwide, and the incidence has tripled over the past four decades [1]. As excess body weight is the consequence of a long term positive energy balance [2], food choices and intake play a central role in the multifactorial nature of obesity [3]. Myriad factors influence what and how much people choose to consume, including biology, psychological factors, and the external environment [4]. From a biological standpoint, taste has long been considered to have a powerful impact on eating behaviour [5]. In that sense, humans preferentially eat what we like [6], probably eat more of what we like [7], and definitely do not eat what we do not like [8]. This seemingly simple observation involves inputs from different systems, and the final hedonic decision integrates metabolic needs with activity in the brain’s reward regions [9].

The strong affective and rewarding appeal of sweet taste may be a primary reason why sweet-tasting foods and drinks are eaten in excess, independent of the body’s need for energy. Specifically, upon arrival of the sweetness-specific afferent signal from the periphery (e.g., mouth, gut) to the brain, dopaminergic pathways are activated causing an increased release of striatal dopamine [10] that is known to mediate the rewarding effects of food ingestion [11]. Operating in concert with dopaminergic neurons [12], a role of opioids in signaling the hedonic pleasure elicited from sweetness has been strongly supported in both the animal [13] and human literature e.g., in [14,15]. Indeed, according to Berridge’s model, dopamine projections to the mesolimbic system are thought to determine the motivation to eat, while the opioid-dependent hedonic hotspots in nucleus accumbens and ventral pallidum contribute to the formation of the affective pleasure of the taste stimulus [16,17]. New insights into gut–brain communication also suggest post oral detection of caloric sugars may generate an additional positive feedback mechanism (‘appetition’) which is thought to stimulate liking and intake for carbohydrate-rich foods by activating dopamine reward systems in the brain and possibly other neurochemical and/or hormonal systems [18,19]. All the above may, in turn, promote overconsumption of sweet tasting foods and drinks beyond energy needs. These putative biological mechanisms are supported by epidemiological data showing that daily intake of sugars frequently exceeds recommendations [20]. Elsewhere, a 2019 review on relationships between sweetness and dietary choices proposed that, unlike taste sensitivity or perception, liking for ever higher sweetness may serve as a good predictor of intake of sweet-tasting foods and drinks [21].

Collectively then, if food choices contribute to obesity, and affective taste responses govern dietary intake, liking for high levels of sweetness may be a potential driver for obesity. Despite a longstanding belief that this is true by both researchers and the public, empirical evidence that intake of sugars or strong pleasure from sweetness contributes to obesity is lacking. Some have put forth the argument that use of sugars has noticeably increased since the 1970s alongside obesity rates [22]; further, given their association with unhealthy eating habits, simple/added sugars, along with fats and salt/sodium, are key dietary components targeted for reduction in the Western diet [23]. However, modern data indicate prevalence of obesity continues to rise despite a drop in intake of simple sugars and sugar-sweetened beverages in both the US [24] and Australia [25]. Moreover, systematic reviews and meta-analyses of controlled trials have shown simple sugars do not behave any differently from other macronutrients in driving weight gain [26]. Still, in alignment with evidence that liquid calories are less filling and induce poor energy compensation compared to solid foods [27], overconsumption of sugar sweetened beverages in specific has been associated with adverse effects of sugar intake beyond calories e.g., in [28].

Critically for the present context, data from studies on affective responses to sweetness have had inconsistent findings with regard to obesity. Some studies report no significant relationship [29,30,31,32,33,34,35,36,37] while others suggest individuals with overweight or obesity experience less pleasure from high sweetness compared to normal-weight individuals [38,39,40,41,42,43]. To better understand reasons for these conflicting reports, a critical consideration is the classification methods used to identify distinct sweet-liking patterns (i.e., sweet-liking phenotypes). Research in the UK [44,45], the US [31], and Korea [46] have all found evidence that liking for sweet taste can be separated into three distinct and definable phenotypes: those expressing strong liking to high levels of sweetness (sweet likers; SLs), those who have aversive responses to strong sweet tastes (sweet dislikers; SDs), and a third group exhibiting maximum liking for a moderate concentration of sucrose [47]. Prior to this emerging consensus, there was a major lack of agreement in criteria used to identify these patterns of hedonic responses across studies [47], leading to an overly simplistic dichotic classification (SLs versus SDs) which failed to adequately describe the full range of human behavioural responses to sweetness. Furthermore, earlier studies had strong potential for misclassification, as the same individual might have been identified as a SD by one method but not with another method. Consequently, it has been difficult to achieve consensus on whether individual differences in liking for sweetness are in fact a risk factor for overconsumption, weight gain, or obesity. These concerns were recently raised by Tan and Tucker [21] in their review on the influence of sweet-liking on food choice and intake. They concluded the use of sweet-liking phenotypes, as opposed to treating affective responses as a continuous measure, will be central in elucidating effects of liking for intense sweetness [21].

A separate limitation in evaluating the influence of individual differences in sweet-liking as potential drivers of obesity comes from an overreliance on body mass index (BMI). As BMI fails to differentiate between body tissues, it is a crude estimate of body composition, particularly for values below 30 kg/m^2^; indeed, half of individuals not labelled as overweight or obese may still have excess adiposity [48]. Notably, studies using BMI diverse samples identify participants with obesity more often as SDs compared to those with normal-weight [38,39,40,41,43]. Conversely, datasets with truncated BMI ranges (mean BMI between 20.1 kg/m^2^ and 27.2 kg/m^2^) have either failed to find an effect of the sweet-liking phenotype on BMI [30,31,32,35,49,50,51] or differences in BMI between phenotypes which failed to reach statistical significance [29,33,36,37]. To date, only one study in adults has investigated body composition as a function of sweet-liking, and they found no evidence of differences in body fat across liker phenotypes [31].

In summary, given the widespread assumption that sugar intake is a driver of obesity, the lack of clarity in prior work suggests targeted data to clarify these issues are warranted. Specifically, two key issues need to be addressed. First, earlier studies most used overly-simplistic classification methods that lacked statistical validity and inflated the likelihood of misclassification: the emergence of a better defined method for defining phenotypes [47] can be used toward more robust evaluation of these relationships. Second, most prior studies have focused on samples from a single country, thereby ignoring the importance of cross-cultural differences in obesity aetiology [52]. Here, we address this gap by testing our hypotheses in two countries with different levels of exposure to an obesogenic environment (the UK and the US). To complement our primary focus on body composition, we also included some common behavioural measures, of lifestyle and dietary characteristics to gain additional insight into differences in eating behaviour between sweet-liking phenotypes that may help explain potential differences in body composition.

## 2. Materials and Methods

### 2.1. Participants

Adults aged 18–34 years were locally recruited from the University of Sussex (UK cohort) and the Pennsylvania State University (US Cohort) to take part in a two-session lab-based study (UK: September to December 2017; US: October to November 2018) advertised as a ‘Taste and Body Metabolism’ study. To qualify for the study, participants were required to be free of medication (other than oral contraceptives), non-smokers (less than five cigarettes a week), and without a history of diagnosed eating disorders, and to report a regular menstrual cycle if a woman. Individuals currently dieting or suffering from a respiratory illness, and who had undergone a dental procedure in the two weeks prior to testing were excluded. On arrival at the research facilities, written informed consent was obtained, but participants remained naive to the study’s hypotheses until they completed all tasks. The University of Sussex Science and Technology Cross-Schools Research Ethics Committee in the UK (ER/VI40/1) and the Penn State Institutional Review Board in the US (STUDY00010753) approved all testing procedures (22 September 2017 and 17 October 2018, respectively). The study was conducted according to the guidelines of the Declaration of Helsinki.

### 2.2. Sensory Measures

Participants evaluated liking (visual analogue scales) and intensity (generalised labelled magnitude scales) for 7 suprathreshold sucrose concentrations prepared based on water and a water blank solution ranging from 0 to 1 M (0.0, 10.7, 21.4, 42.8, 85.6, 171.2, 228.2, and 342.3 g sucrose per 1 L solution). Aliquots of 10 mL of each stimulus were presented at room temperature in a randomised order using a sip and spit protocol. The taste test was replicated in two separate blocks for a total of 16 tastings. Participants were advised to refrain from eating and drinking flavoured beverages for the two hours prior to the taste test. Pre- and post-test levels of hunger, satiety, and thirst were also recorded. More details about the sweet taste test can be found in Iatridi et al. (2019) [44]. All ratings in the UK cohort were collected using the Sussex Ingestion Pattern Monitor (SIPM version 2.0.13, University of Sussex, Falmer, UK); all ratings for the US cohort were collected using Compusense Cloud, Academic Consortium (Guelph, ON, Canada).

### 2.3. Anthropometric Measures

In both cohorts, all anthropometric assessments took place on a second visit, and were always conducted by the same trained researcher. Standing height to the nearest 0.1 cm using a wall stadiometer and body weight to the nearest 0.1 kg using the electrical weighing scales integrated into the bioelectrical impedance devices listed below were taken. Standard procedures were followed, including wearing light clothing but no shoes [53]. Waist circumference and hip circumference were measured in duplicate to the nearest 0.5 cm with a stretch-resistant tape [54] and means were used for analysis. Total body fat (BodyFat) and fat-free mass (FFM) were evaluated from body composition measures assessed using a multi-frequency segmental bio-impedance device (MC-780MA P, TANITA, UK and BC-418, TANITA, US). Given that bioelectrical impedance analysis (BIA) relies on specific assumptions, including body hydration status, specific instructions were provided to all participants prior to the experimental day [55]. Specifically, participants were asked to refrain from consuming alcohol for 24 h and from performing strenuous exercise for 12 h before the anthropometry session. Appointments were scheduled at between 0700 and 1030 h after an 8-h fast and water abstinence; participants were also advised to avoid having a long shower or a bath on that morning. Body composition measures were taken whilst the participant had bare feet and carried no metal objects, were wearing light clothing, and after using the bathroom facilities in the laboratory.

### 2.4. Demographic, Lifestyle, Behavioural, and Dietary Characteristics

Participants provided information about demographic characteristics (date of birth, sex, ethnicity), dieting (i.e., being a former, current, or never-dieter), breakfast habits, and sleeping routine (i.e., bedtime, wake-up time, and midday naps separately for weekdays and weekends). To assess physical activity level, the short form of the International Physical Activity questionnaire was administered [56]; based on its scoring algorithm [57], this questionnaire allows participants to be classified into low, moderate, and high physical activity groups.

Standard questionnaires assessing personality traits related to eating behaviour were also administered. The behavioural questionnaires administered included the original 51-item Three Factor Eating Questionnaire (TFEQ) [58] which presents questions about restrained eating, which is defined as the tendency to consciously restrict food intake in order to control body weight, disinhibition that concerns loss of control over eating in response to negative emotions or the presence of highly palatable foods, and trait hunger which is designed to measure the extent to which hunger feelings are perceived and drive food intake. From the Barratt Impulsiveness Scale [59] that assesses the predisposition to react to internal or external stimuli without adequate forethought about the consequences that are favoring immediate rewards over long-term goals, we examined the attentional, motor and non-planning impulsiveness subtypes. Participants were also asked to complete the English language version [60] of the Sensitivity to Punishment and Sensitivity to Reward Questionnaire [61]. The Sensitivity to Punishment subscale refers to the behavioural inhibition under specific conditions of threat, punishment or non-reward, whereas the Sensitivity to Reward subscale reflects approach behaviour to specific conditioned and unconditioned rewards, appetitive stimuli included. Finally, responses on the Arnett’s Inventory of Sensation Seeking questionnaire [62] were obtained. Adapted from the original Zuckerman’s Sensation Seeking Scale [63], this questionnaire examines different constructs of sensation seeking (e.g., thrill, adventure, and experience seeking, boredom susceptibility etc.) that may be captured through two subscales: intensity seeking and novelty seeking. For all questionnaires listed above, higher scores indicated a more significant presence of the personality trait under investigation.

For the dietary assessment, the European Prospective Investigation of Cancer (EPIC) Norfolk Food Frequency Questionnaire (FFQ) [64] adapted to incorporate a more extended list of beverages (energy drinks, sweetened canned tea, and concentrated juice drinks without added sugar), and the 15-item Beverage Intake Questionnaire [65] were completed by participants in the UK and the US, respectively. Dietary data were accordingly transformed to continuous measures prior to analysis (see 2.5 for details).

### 2.5. Statistical Analysis

Consistent with contemporary best practices, agglomerative hierarchical cluster analysis with squared Euclidean distance and the average linkage method was used for the identification of distinct patterns of liking for increasing sweetness (sweet-liking phenotypes). Clustering was performed on the mean liking ratings from the eight replicated stimuli concentrations, and the decision on the final number of clusters was informed by the magnitude of the difference between the coefficients of the agglomeration schedule and application of this information to the dendrogram produced as part of the statistical output [66]. To eliminate overfitting, two-by-two cross-tabulation were implemented aiming at reclassification of roughly 7% of each cohort’s sample showing non-erratic atypical hedonic responses through identifying the dyads of sucrose concentration and liking scores with the highest sensitivity and specificity in predicting the three sweet-liking phenotypes; more details on the clustering approach can be found in Iatridi et al. (2019) [44].

Except for age and BMI, which are expressed as medians and 25th and 75th percentiles and later log-transformed to improve normality, means and standard errors of the means (SEMs) are used throughout; categorical variables are shown as percentages. One-way analysis of variance (ANOVA) or, when sex and/or age were included as covariate (s), one-way analysis of covariance (ANCOVA) were used. Fisher’s least significant difference was used as the post hoc test and Welch tests and Games–Howell follow-up analysis was applied when equal variance assumptions were violated. Additionally, between subjects two-way (country or age group by phenotype) ANOVAs or ANCOVAs were carried out to determine if there were significant differences in the measured outcomes, while the interaction effect between sucrose concertation and phenotype or country was analyzed with two-way Repeated Measures ANOVAs with Greenhouse–Geisser correction in cases of violation of assumption of sphericity. Eta squared values (*ηp*^2^) are reported as the measure of effect sizes for the main analyses and were considered small when equal to 0.01, medium when equal to 0.06 and large when equal to 0.14. Finally, to quantify differences in each of the obesity-related anthropometric measures by phenotype when habitual intake of sweet-tasting beverages was accounted for, multiple linear regression models with dummy coding were employed. Variance inflation factors were used to check for multicollinearity in our models with more than one predictor; no evidence of multicollinearity was observed.

Student’s t-tests (continues outcomes) and Pearson’s chi-squares (categorical outcomes) were used to compare the various demographic, lifestyle, behavioural, and dietary data between cohorts and between age groups. Degrees of freedom were adjusted as appropriate when equal variances were not assumed. For the semi-quantified food frequency questionnaires, in order to facilitate direct comparisons of beverage use between cohorts and across taste phenotypes the 9- and 7-point frequency consumption scales in the UK- and the US-specific questionnaires, respectively, were transformed as an annualized estimate of intake e.g., 1/week = 52, 1–3/month = 104, etc., [67]. Additionally, to control for differences in portions between the two food frequency questionnaires, frequency x portion was calculated for the Beverage Questionnaire-15 and was further reduced to the portion reported on the EPIC Norfolk food frequency questionnaire (e.g., 1/day × 12 ounces of soft drink = 365 × 1.5 glasses of soft drink). To reduce skew in annualized intake data, values were log_e_ transformed.

Anthropometric data were not available for three participants (two women and one man) in the UK and ten participants (eight women and two men) in the US who failed to return for session 2. Moreover, four participants, two from each cohort, provided contradictory information about their dieting history at pre-screening and the first day of testing, so they were excluded from analyses related to anthropometrics, lifestyle and eating habits, and eating behaviour. Significance was set at *p* < 0.05. All statistical calculations were performed using IBM SPSS Statistics for Windows, version 25.2 (Chicago, US).

## 3. Results

A total of 148 participants in the UK (29.1% men; 75.7% Caucasians) and 126 in the US (32.5% men; 81.7% Caucasians) completed the taste test and the behavioural (Table S1), lifestyle (Table S2), and dietary questionnaires (Table 2). 145 participants in the UK and 116 participants in the US also attended the separate anthropometric session (Table S3). Despite recruitment in the same age range, participants in the UK were slightly, but significantly, younger than those in the US (UK: *Mdn* = 20.2 years vs. US: *Mdn* = 22.0 years; *t*(184.306) = −3.323, *p* = 0.001); accordingly, the effects of age on between-country findings were considered in subsequent analyses.

### 3.1. Identification of Distinct Sweet-Liking Phenotypes

**Figure 1:**
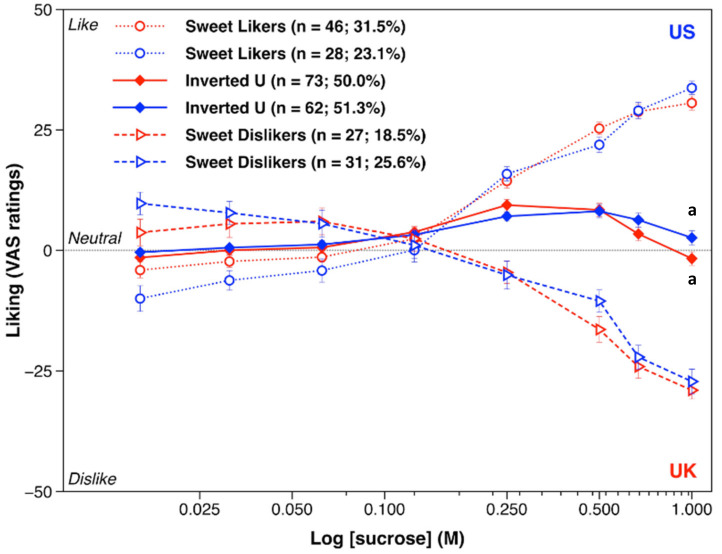
Liking ratings (mean ±standard error of the mean) as a function of sucrose solutions by the three sweet-liking phenotypes by cohort. Ratings were averaged across the two taste test blocks prior to clustering. Blue and red features represent ratings recorded in the US and the UK, respectively. The response pattern for the sweet-liking phenotype is displayed with a dotted line, the response pattern of the inverted U-shaped phenotype with a solid line, and the response pattern of the sweet disliker phenotype with a dashed line. Liking ratings within a sweet-liking phenotype that share a letter significantly differ.

As shown in Figure 1, hierarchical cluster analysis revealed three main distinct hedonic response patterns to sweet taste: a sweet liker phenotype (SL) showing a rise in liking with increasing sucrose concentration, a sweet disliker phenotype (SD) characterized by a decline in liking as sucrose concentration increased, and an inverted-U group (IU) where participants expressed optimal sweetness at either 0.25 M or 0.5 M sucrose; liking ratings for the 0.25 M sucrose in the UK (*M* = 9.42, *SEM* = 1.172) and the 0.5 M sucrose in the US (*M* = 8.12, *SEM* = 1.377) did not significantly differ between cohorts (*t*(133) = 0.728, *p* = 0.468). Analysis of variance (ANOVA) confirmed the effect of phenotype on overall liking for each cohort (UK: *F*(2, 143) = 116.41, *p* < 0.001, *ηp*^2^ = 0.619; US: *F*(2, 118) = 37.15, *p* < 0.001, *ηp*^2^ = 0.386). Phenotypic differences in overall liking remained significant after controlling for pre-test levels of hunger and thirst.

When each phenotype was examined separately, participants in the UK and in the US shared very similar hedonic response patterns. In particular, all within-phenotypes between-cohorts contrasts per sucrose concentration were non-significant save one: participants in the UK cohort who were classified into the inverted-U group liked 1 M sucrose solution more than the comparable sub-group in the US cohort (UK: *M* = −1.74, *SEM* = 1.389; US: *M* = 2.60, *SEM* = 1.480; *t*(133) = −2.137, *p* = 0.034). Finally, the prediction value of the +15/−15 cut-off liking scores on the −50 to +50 visual analogue scale (SL: 97.7 percentage sensitivity and 93.5 percentage specificity for the +15 liking score; SD: 90.9 percentage sensitivity and 93.9 percentage specificity for the −15 liking score; interclass correlation coefficient: 0.763 95%CI (0.662, 0.832)) alongside the good reproducibility of the 1 M sucrose reported in Iatridi et al. (2019) [44] for the UK cohort were confirmed in the US sample, too (SL: 96.3 percentage sensitivity and 95.3 percentage specificity for the +15 liking score; SD: 92.3 percentage sensitivity and 95.4 percentage specificity for the −15 liking score; interclass correlation coefficient: 0.881 95%CI (0.820, 0.920)).

### 3.2. Effect of Phenotype and Country on Participant Characteristics

#### 3.2.1. Demographics

**Figure 2:**
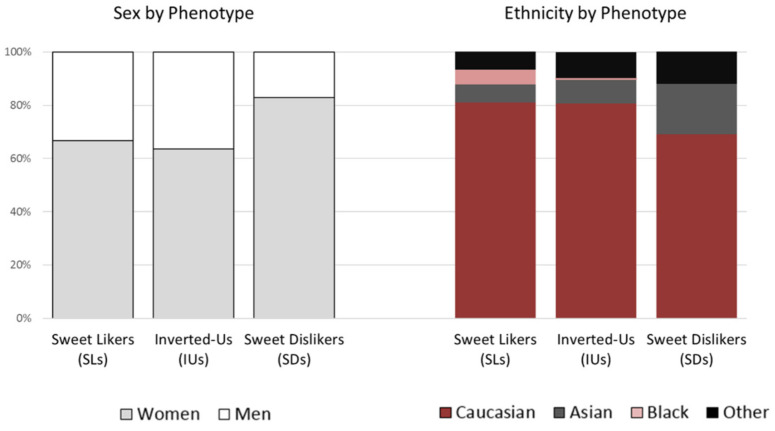
Proportion (%) of sexes and ethnicities by phenotype.

Across phenotypes, sex (*χ*^2^(2, *n* = 267) = 6.541, *p* = 0.038) and ethnicity (*χ*^2^(6, *n* = 267) = 14.050, *p* = 0.029) were significantly different. As shown in Figure 2, men were more often SLs than SDs, while participants self-identified as Asians were twice as likely to be SDs as SLs; for Caucasians, the IU phenotype was the most prevalent, followed by the SL and the SD phenotype. ANOVA showed no effect of phenotype on age (*F*(2, 264) = 0.863, *p* = 0.423).

#### 3.2.2. Anthropometry

Age was found to contribute significantly to the regression models predicting each anthropometric measure by phenotype with all associated changes in *F*-values being significant (Table S4). To further explore this moderating hypothesis, a median split on age was used to categorize participants into younger and older groups. A phenotype by age group interaction was found for BMI (*F*(2, 244) = 3.034, *p* = 0.050, *ηp*^2^ = 0.024), as well as for BodyFat (*F*(2, 243) = 3.506, *p* = 0.032, *ηp*^2^ = 0.028), FFM (*F*(2, 243) = 4.315, *p* = 0.014, *ηp*^2^ = 0.034), waist circumference (*F*(2, 243) = 3.413, *p* = 0.035, *ηp*^2^ = 0.027), and waist to hip ratio (*F*(2, 243) = 2.764, *p = 0*.065, *ηp*^2^ = 0.022), in models adjusting for sex. In plotting these interactions, it was apparent that the effect of the sweet-liking phenotype on the anthropometric measures was in opposite directions for participants who were younger than 21 years old or relatively older in these samples. In contrast, there was no evidence of interactions between country and phenotype for any of the anthropometric measures under investigation: BMI (*F*(2, 244) = 2.186, *p* = 0.115), BodyFat (*F*(2, 243) = 2.340, *p* = 0.098), FFM (*F*(2, 243) = 0.623, *p* = 0.537), waist circumference (*F*(2, 243) = 1.997, *p* = 0.138), and waist to hip ratio (*F*(2, 243) = 1.087, *p* = 0.339).

**Figure 3:**
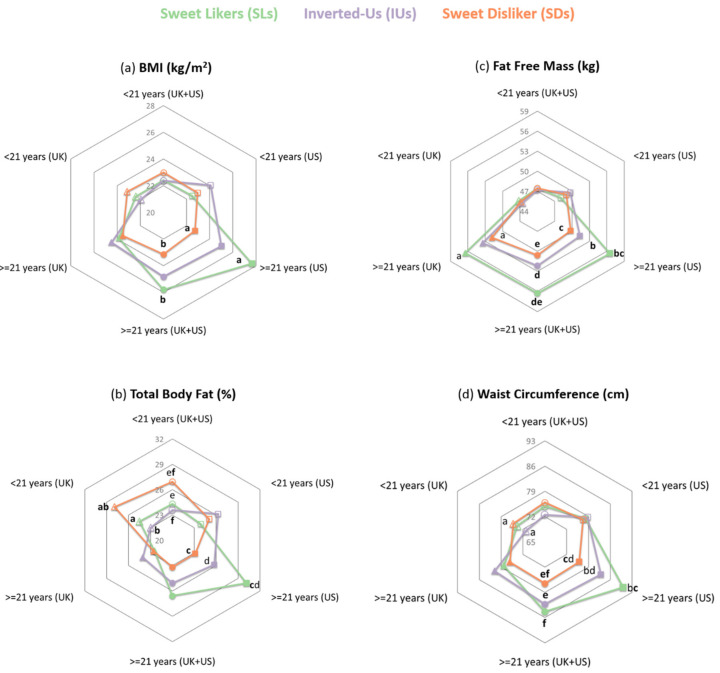
A comparison of the follow-up effects of the sweet liker phenotype on the anthropometric profile for the two cohorts separately and for the sample as a whole (UK + US) plotted by age group. Spider plots show the estimated marginal mean values oF(**a**) BMI (kg/m2) and oF(**b**) Total Body Fat (%), (**c**) Fat Free Mass (kg), and (**d**) Waist Circumference (cm) adjusted for sex. Examining each spider plot separately, the same letters indicate significant differences (*p* < 0.05; bold) or tendencies (*p* < 0.075) in the paired post hoc comparisons of the estimated marginal mean values of each anthropometric measure of interest. For example, among participants 21 years old and older of both cohorts (UK + US), sweet dislikers (SDs) had lower BMI (23.1 kg/m^2^) than sweet likers (SLs) (25.8 kg/m^2^; *p* = 0.016), but did not significantly differ in their mean BMI values from participants classified into the inverted-U (IU) phenotype (24.8 kg/m^2^; *p* = 0.082). For BMI, highlighted *p*-values correspond to analysis of variance of BMI’s natural logarithm; original BMI values are used for graphical representation only. BMI, body mass index.

As shown in Figure 3c, in participants aged 21 years and older, post hoc analyses showed that SLs had (US cohort) or tended to have (UK cohort) greater FFM when compared to SDs (US: *M* = 56.55 kg, *SEM* = 1.72 kg for SLs vs. *M* = 49.76 kg, *SEM* = 1.48 kg for SDs; *p* = 0.005; UK: *M* = 56.44 kg, *SEM* = 1.49 kg for SLs vs. *M* = 51.84 kg, *SEM* = 1.79 for SDs; *p* = 0.056; all comparisons accounted for sex), FFM was also greater in SLs relative to IUs, but significance was achieved only in the US cohort (*p* = 0.010). Critically, despite that, in the UK cohort, those 21 years old and older comprised only 33% of the sample, models for the effect of phenotype on FFM revealed medium to large effect sizes in both countries (UK: *ηp*^2^ = 0.094; US: *ηp*^2^ = 0.111; Table 1). Observed power was estimated at 42 and 77% for the subgroup analysis of the UK and the US samples, respectively. Regarding phenotypic differences in obesity-related measures (Figure 3a,b,d), while older SLs appeared to have worse anthropometric profiles relative to SDs in both cohorts, this was confirmed statistically only in the US participants. In particular, older SLs in the US cohort had higher BMIs by 4.3 kg/m^2^, BodyFat by 7.0%, waist circumference by 14.11 cm, and waist to hip ratio by 0.07 (BMI: *p* = 0.007; BodyFat: *p* = 0.010; waist circumference: *p* = 0.003; waist to hip ratio: *p* = 0.025) when compared to the SDs.

As expected from the interaction effect of age group on the relationship between sweet-liking phenotypes and anthropometric measures, SDs younger than 21 years old recruited in the UK had significantly higher BodyFat (*M* = 27.9%, *SEM* = 1.3%) than the SLs (*M* = 24.5%, *SEM* = 0.9%, *p* = 0.030) and the IUs (*M* = 23.0%, *SEM* = 0.8%, *p* = 0.001) of the same age group. In contrast, this was not seen for the younger US participants: despite SDs having higher BodyFat by 1.16 and 1.22 percentage units when compared with SL or IU phenotypes, respectively, the post hoc tests were not significant. The low representation of the younger age group in the US cohort (27.5%) may have reduced statistical power from 84% for the relevant ANCOVAs in the UK to 8% in the US. Indeed, when the effect of phenotype on BodyFat was examined in the two populations combined, a significant effect of phenotype was found (*F*(2, 121) = 3.062, *p* = 0.050, *ηp*^2^ = 0.048).

Examining the phenotypic differences in anthropometry by sex, the same age-specific trends were revealed. To note, due to the low representation of men particularly in the younger age group (SL: *n* = 9; 21%, IU: *n* = 15, 25%, SD: *n* = 3, 11% in the <21 year old group; SL: *n* = 18, 58%, IU: *n* = 28, 39%, SD: *n* = 7, 23% in the ≥21 year old group), some ANOVAs by sex and age group were not sufficiently powered to reach significance (*p* < 0.05) or show a tendency (*p* < 0.075). Specifically, in participants 21 years old and older, an effect of phenotype on FFM was evident in men (*F*(2, 49) = 4.177, *p* = 0.021, *ηp*^2^ = 0.146), but failed to reach significance in women (*F*(2, 70) = 2.199, *p* = 0.119, *ηp*^2^ = 0.059). Notably, most post hoc comparisons revealed that both SL men and SL women had higher FFM (men: *M* = 67.0 kg, *SEM* = 2.2 kg; women: *M* = 48.0 kg, *SEM* = 0.9 kg) than both SDs (men: *M* = 58.3 kg, *SEM* = 1.1 kg, *p* = 0.010; women: *M* = 44.1 kg, *SEM* = 1.1 kg, *p* = 0.044) and IUs (men: *M* = 62.3 kg, *SEM* = 1.2 kg, *p* = 0.039; women: *M* = 44.9 kg, *SEM* = 0.8 kg, *p* = 0.079). ANOVAs and follow-up analyses for the effect of phenotype on BMI and waist circumference that were performed separately for men and women 21 years old and older, confirmed the trends reported for the sample as a whole. However, significant differences were observed only in men (BMI: *F*(2, 49) = 3.254, *p* = 0.047, *ηp*^2^ = 0.117; waist circumference: *F*(2, 49) = 3.290, *p* = 0.046, *ηp*^2^ = 0.118). Regarding the phenotypic differences in BodyFat among participants younger than 21 years old, although BodyFat was measured to be higher in both SD men (*M* = 17.4%, *SEM* = 4.8%) and SD women (*M* = 29.3%, *SEM* = 1.1%) compared to SL men (*M* = 15.9%, *SEM* = 1.1%) and SL women (*M* = 26.4%, *SEM* = 1.1%), respectively, a tendency towards an overall effect of phenotype on BodyFat was calculated only in the subgroup of women (*F*(2, 97) = 2.738, *p* = 0.070, *ηp*^2^ = 0.053).

#### 3.2.3. Behaviour, Lifestyle, and Diet

Analysis so far suggests hedonic responses to sweetness may reflect body composition and/or sizes, but this occurs in an age-specific manner. To interpret these observations, contributions of behaviour, lifestyle, and diet were considered.

First, the younger and older groups reflected shorter and longer periods of exposure to the obesogenic environment: participants younger than 21 years old scored lower in restraint eating (*t* (261) = −2.471, *p* = 0.014), reported sleeping 31 min more per day (*t* (216.84) = 3.643, *p* < 0.001) and were non-dieters more often (*χ*^2^(1, *n* = 263) = 3.526, *p* = 0.060) relative to participants aged 21 years or older. Behavioural, lifestyle, and dietary characteristics of US participants also reflected a higher exposure to the obesogenic environment relative to UK participants. In particular, a higher proportion of the US cohort regularly skipped breakfast (US: 14.5%; UK: 4.8%; *χ*^2^(2, *n* = 270) = 7.622, *p* = 0.022) and reported a shorter sleep duration than the UK cohort (US: *M* = 8.29 h/day, *SEM* = 0.075 h/day; UK: *M* = 8.84 h/day, *SEM* = 0.104 h/day; *t* (265) = −4.147, *p* < 0.001); differences remained significant after controlling for age. The US cohort was also characterized by higher dietary restraint (*F*(1, 267) = 7.097, *p* = 0.008, *ηp*^2^ = 0.026) and lower scores on the TFEQ-hunger scale (*F*(1, 267) = 4.066, *p* = 0.045, *ηp*^2^ = 0.015), lower attentional and non-planning impulsivity (*F*(1, 267) = 4.973, *p* = 0.027, *ηp*^2^ = 0.018 and *F*(1, 267) = 19.847, *p* < 0.001, *ηp*^2^ = 0.069, respectively) and weaker seeking for intensity and novelty in life experiences (*F*(1, 267) = 3.810, *p* = 0.052, *ηp*^2^ = 0.014 and *F*(1, 267) = 12.078, *p* = 0.001, *ηp*^2^ = 0.043, respectively), independently of age.

**Figure 4:**
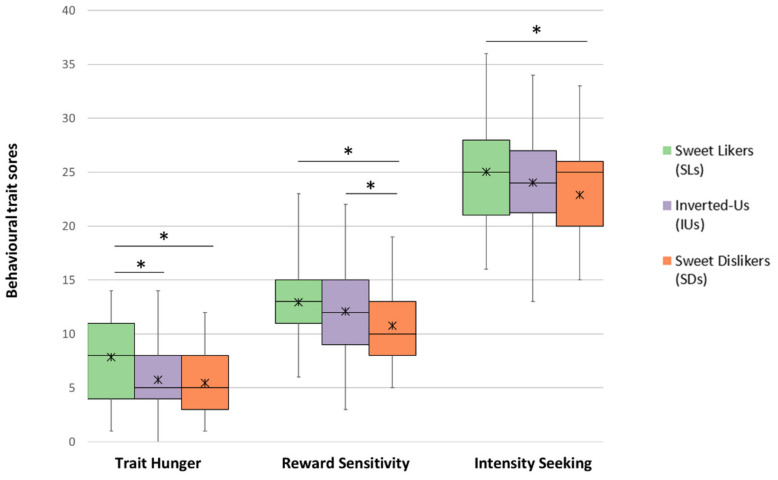
A comparison of selected behavioural traits across the three distinct sweet-liking phenotypes. Boxplots compare the mean scores (🞷 symbol in each box) of trait hunger, reward sensitivity, and intensity seeking by phenotype across the entire sample (UK and US cohort combined). Boxes are the interquartile ranges, whiskers represent the minimum and maximum score of each behavioural trait, and solid lines indicate the medians. Significant differences (*p* < 0.05) in the paired post hoc comparisons are denoted with an asterisk (∗).

Regarding participant characteristics by phenotype, no phenotypic differences in lifestyle habits were found (breakfast skipping: *χ*^2^(4, *n* = 263) = 1.873, *p* = 0.759; sleeping duration: *F*(1, 257) = 0.929, *p* = 0.396; dieting: *χ*^2^(2, *n* = 263) = 2.338, *p* = 0.311; physical activity level: *χ*^2^(4, *n* = 263) = 2.809, *p* = 0.590). Conversely, relevant ANOVAs for behavioural characteristics, revealed significant effects of phenotype on reward sensitivity (*F*(2, 260) = 5.616, *p* = 0.004, *ηp*^2^ = 0.041), intensity seeking (*F*(2, 260) = 4.163, *p* = 0.017, *ηp*^2^ = 0.031), and TFEQ-hunger (*F*(2, 260) = 11.705, *p* < 0.001, *ηp*^2^ = 0.083). Post hoc tests revealed that SLs scored higher on these three behavioural subscales than SDs did, whilst contrasts between SLs and IUs were only significant for TFEQ-hunger, i.e., trait hunger (Figure 4). Critically, SLs maintained these elevated values across age groups (TFEQ-hunger: *t*(72) = 0.025, *p* = 0.980; intensity seeking: *t*(72) = 0.489, *p* = 0.627). Likewise, no interaction effect of age group neither of country on the phenotypic differences in trait hunger (phenotype x age group: *F*(2, 257) = 0.850, *p* = 0.428; phenotype x country: *F*(2, 257) = 0.450, *p* = 0.638) and intensity seeking (phenotype x age group: *F*(2, 257) = 0.787, *p* = 0.457; phenotype x country: *F*(2, 257) = 0.810, *p* = 0.446) were observed. For reward sensitivity, age group strongly interacted with phenotype for reward sensitivity (*F*(2, 257) = 8.562, *p* < 0.001, *ηp*^2^ = 0.062) with higher values recorded in our older subgroup; a parallel interaction was not seen for country (*F*(2, 257) = 0.971, *p* = 0.380).

To test a possible role of diet on observed relationships between sweet-liking patterns and anthropometry, we examined self-reported use of beverages. Due to the difference in the legal drinking age in the UK and the US, analyses related to habitual intake of alcoholic drinks were restricted to participants 21 years old and older. Country- and phenotypic differences in beverage habitual intake are shown in Table 2.

To identify dietary predictors that significantly improved fit of anthropometry-specific regression models, multiple linear regression with forced entry was used in the sub-cohorts and for the anthropometric measures that phenotypic differences had emerged (Figure 3a–d, Table 1). After dummy variable transformations using the SL phenotype as the baseline group against which the IU and SD groups would be compared, adding the frequency of beverage intake of younger participants to prediction models of BodyFat resulted in an increase in F statistic by just 0.187 (*p* = 0.992) and 0.472 (*p* = 0.796) for the UK subgroup and the entire sample younger than 21 years old, respectively; no diet-related predictors emerged. From this, we can conclude relationships between phenotype and BodyFat for participants younger than 21 years old did not significantly change as a function of beverage intake. Conversely, among participants 21 years old and older tested in the US, sweetened fruit beverages (e.g., concentrated juice drinks, juice drinks with added sugar) were a significant predictor of BMI (*β* = 0.248, 95% CI (0.000, 0.041), *t* = 2.012, *p* = 0.048, *R*^2^ = 0.325) and waist circumference (*β* = 0.300, 95% CI (0.926, 0.7.571), *t* = 2.552, *p* = 0.013, *R*^2^ = 0.248) with the models including the dietary factors explaining 15.7% and 20.2% additional variance in BMI and waist circumference between phenotypes; furthermore, frequency of intake of soft drinks tended to predict BodyFat, (*p* = 0.057 and *p* = 0.054 for regular and diet soft drinks, respectively).

## 4. Discussion

### 4.1. General Findings

In both cohorts tested, we confirmed the existence of three distinct hedonic response patterns to stimuli of varied sweetness: the SL, inverted U, and SD phenotypes. Regarding the link between sweet-liking and weight status or body composition, our data fail to support a simple model where sweet-liking always leads to obesity. In fact, we provide novel evidence that FFM is potentially the main anthropometric measure involved in the pattern of hedonic response to sweetness. Further, our data suggest that the effect of phenotype on body composition varies with age. In the younger group, SDs presented with the highest BodyFat, whereas for the older subgroup, SLs had higher BMI, waist circumference, and FFM. Here, increased age appeared to reflect behavioural and lifestyle indices typical of increased exposure to an obesogenic environment. Intake of sweet-tasting beverages partially mediated the phenotypic differences in obesity-related anthropometric measures, but only in the older subgroups (i.e., those with longer exposure to an obesogenic environment). Finally, we identified behavioural characteristics that may explain the phenotypic differences in anthropometrics: SLs had enhanced sensitivity to rewarding stimuli and characteristics analogous to those of high interoceptive performers.

### 4.2. What Do Sweet-Liking Patterns Can Tell Us about Individual Variation in Anthropometry?

To the best of our knowledge, this is the first study to consider a role of FFM in taste hedonics. Highlighting a potentially important determinant of the link between level of liking for sweet taste and anthropometry, here, we report a strong effect of the sweet-liking phenotype on FFM in participants 21 years and older. Furthermore, consistent results from both cohorts indicated that the greatest FFM was observed in participants classified as SLs.

Regarding the mechanisms underlying these effects, they might be rooted in biology. For example, to maintain energy balance, FFM with its known contribution to daily energy requirements [68], exerts orexigenic effects and thus promotes energy intake, as opposed to fat mass, which may have an inhibitory role in appetitive control [69]. Specific adaptations in eating behaviour/patterns consistent with ensuring higher energy intake such as larger self-determined meal sizes [70] or higher eating rate [71] have been positively associated with FFM; such links have been absent for BodyFat and/or BMI. Consistent with the idea that the body is tuned to prioritize signals deriving from FFM over those from fat mass, FFM has also been suggested to relate to neuronal density in brain areas involved in homeostatic regulation and eating behaviour independently of fat mass [72]. The phenomenon of collateral adiposity or simply ‘fat overshooting’, where the potent internal signal for recovery of FFM after weight loss induces overeating and consequently a disproportional increase in fat mass, further emphasizes the critical importance of FFM over fat mass in regulating energy intake [73]. Recently, disliking for low sweetness was proposed to be positively associated with habitual exercise levels [74]. Given that, in the absence of differences in BMI or age, active individuals are expected to have relatively higher FFM than those being more sedentary, it could be theorized that the taste stimulus that signaled the poorest energy content (i.e., the stimulus of low sweetness) was likely to evoke lower liking among more active individuals. Staying with that idea, men have higher levels of FFM compared to women [75], and strikingly, in our data, we find that men were classified as SLs significantly more often than women. Similarly, the loss of FFM and relative increase in fat mass in the absence of changes in BMI that occur with ageing [76] might offer an appealing explanation for the often reported inverse relationship between age and liking for sweetness [31,77].

Here, we also observed significant effects of sweet-liking patterns on multiple obesity-related anthropometric measures. However, the direction of these relationships was not straightforward. Interaction analysis suggested a dissociation of anthropometric measures by phenotype depending on age. That is, for participants younger than 21 years of age, being classified into the SD phenotype was associated with the highest BodyFat percentage, whilst SLs 21 years old and older had significantly higher BMI and waist circumference relative to SDs (Figure 3a–d). Moreover, the IU phenotype had an equally good anthropometric profile to SLs when younger, and only differed from SLs when older, although they were still presented with anthropometric profiles closer to those of SLs than of SDs. It is tempting to speculate that the interaction between age and phenotype found here may provide an explanation as to why a considerable number of studies seeking to describe how sweet-liking patterns relate to obesity have failed to show consistent results.

Before exploring this hypothesis further, we also note many previous attempts to explore influences of individual differences in sweet-liking on obesity have been marred by discrepancies in classification methods, as recently reviewed [47]. Similarly, the singular focus of previous studies on BMI—which is now recognized as a poor predictor of adiposity [48]—may have further obscured potential relationships between phenotype and adiposity; this view is supported by our finding that the effect of phenotype in our lean younger group was seen in differences in BodyFat, but not in BMI. Recently, Garneau et al. [31] also used bioelectrical impedance to assess body composition, and they failed to find any effect of the sweet-liking phenotype on BodyFat. However, it is unclear whether their analyses controlled for participant sex, and under what testing conditions they performed bioelectrical impedance analysis; their data came from a community based sample where hydration status was presumably not controlled, which may have added substantial noise to their estimates of the body composition.

Regarding the role of age in the relation between sweet-liking patterns and obesity, other researchers have also found some age-dependent variation in BMI, although these differences failed to reach significance. For example, in Methven et al. (2016) [33] and Asao et al. (2015) [29], SLs had ~3 units greater BMI than SDs, with participants of a median and mean age of 26 and 32 years, respectively. Presumably, such magnitude of difference in BMI would be clinically meaningful. Yeomans and colleagues [37] analysed a younger cohort and found that SDs were heavier than SLs by 1.4 units. Notably, all of these studies only divided participants into two sweet-liking hedonic response groups. In the NutriNet-Santé cohort, one of the largest ongoing web-based chemosensory studies, liking for natural sweetness expressed as a continuous variable and assessed via an online questionnaire was negatively associated with self-reported BMI in a sample of over 45,000 French adults [77]. Consistent with our age-related observation, those authors noticed that in women, the association between liking scores for all factors composing the sweet sensation and BMI differed by age category: in women 18–34 years old, the higher the liking, the lower the BMI whereas the inverse relationship was proposed for those in the 35–54 and >55 years age groups. However, since liking ratings were not inferred from analyses of lab-based sensory tests and even natural sweetness was referred to food entities of enhanced sweetness (e.g., added jam, honey, gingerbread), some caution should be exercised in interpreting these data. In summary, a close inspection of past research suggests sweet-liking may only drive overconsumption in relatively older adults, while in younger individuals, SL is associated with reduced risk of less healthy anthropometric characteristics.

### 4.3. The Obesogenic Environment Approach

How might age modulate the influence of phenotypic differences in sweet-liking on body composition? One possibility relates to increased exposure to an obesogenic environment over time. Here, the older group scored higher in the TFEQ restrained eating subscale, and they also reported being on a weight loss diet more frequently and sleeping less. These differences in behaviour and lifestyle are likely associated with the obesogenic environment. Restrained eating is thought to be an adaptive behaviour to an environment of oversupply of easily accessible hyper-palatable foods and of the associated cues that amplify temptation in an obesogenic environment [78]. Repetitive dieting is also likely to contribute to disordered eating through predisposing weight gain [79], particularly among normal-weight individuals [80]. Regarding poor sleeping habits, inadequate sleep has been identified as a key feature of modern obesogenic societies [81].

Considering the central role of exposure to an obesogenic environment in our hypothesis, another finding worth highlighting is the age-specific mediating effect of habitual intake of liquid calories and sweetened non-caloric drinks and beverages. Our analysis suggests that with longer exposure to an obesogenic environment, dietary choices are more likely to contribute to a relationship between sweet-liking and anthropometric outcomes. Furthermore, more subtle anthropometric advantages were observed in younger SLs of the US cohort and diminished disadvantages in older SLs of the UK cohort, pointing to possible involvement of the different effects of the obesogenic environment in the two countries. Indeed, between-country differences in restrained eating, sleeping habits, breakfast consumption, and waist circumference were found here. Notably, breakfast skipping is often cited as a component of the modern obesogenic world that may contribute to poor energy regulation [82], and disproportionate abdominal fat is also believed to be a downstream effect of the Western lifestyle [83]. As such, it may be that alongside age-related duration of exposure, the degree of exposure to the obesogenic environment that is linked to cultural-specific factors may also be important.

Critically, studies dating back more than four decades (i.e., to a time when the obesogenic environment was not cast as a public health issue) have found a significant effect of the sweet-liking phenotype on obesity-related characteristics that are consistent with our data in younger participants [38,39,40,41,43]. That is, individuals of normal weight experienced stronger pleasure from high sweetness relative to those overweight or with obesity. In contrast, the more recent literature has failed to show any significant relationships [29,30,31,32,33,34,35,36,37]. Accordingly, we might speculate that recruiting participants of a broad range of ages (i.e., 18–65 years) without accounting for the effect of the exposure to the obesogenic environment may have attenuated links between hedonic responses to sweetness and anthropometric outcomes. For example, in the NutriNet-Santé cohort in France a country lacking the obesogenic profile of the US and the UK where most previous investigations took place, [84], the direction of the relationship between sweet-liking and obesity differed by age group [77]. Collectively, these data suggest the importance of cross-cultural differences must be considered, as they modify the contribution of the external environment to health-related behaviours and outcomes. Clare Llewellyn, the director of the largest twin birth cohort in the UK recently noted: “Somewhat ironically, research into the genetic basis of obesity has revealed more than anything the urgent need for environmental modification.” [85].

### 4.4. The Alliesthesia and Hedonic (Non-Homeostatic) Approach

Above, we provided a framework to show how the obesogenic environment may account for the age-specific effects of sweet-liking on adiposity. However, a conceptual model which can explain observed associations between the distinct sweet-liking phenotypes and anthropometry needs to be elucidated. Our data suggest the answer may lay within SL’s distinctive behavioural profiles, a pattern which is characterized by relatively high values of TFEQ-hunger, intensity seeking, and reward sensitivity (Figure 4). TFEQ-hunger as a proxy of hunger-driven eating and accordingly of better interoceptive abilities was enhanced in our SLs compared to IU and SD phenotypes, and was unaffected by age. Previous studies that examined eating behaviour in relation to sweet-liking only reported non-significant results for restrained and/or disinhibited eating [30,36,37,49]. Meanwhile, SLs here scored higher on the intensity subscale of Arnett’s Inventory of Sensation Seeking (AISS), which could be interpreted as an indirect measure of behavioural adaptation to internal body signals [86]. Robust empirical data linking sensation seeking and hedonics have only recently become available [67,87] and experiments have focused on oral burn from capsaicin. Thus, it is possible to explain this pattern of differences by considering the expression of sweet-liking in relation to homeostasis, as classically suggested by Cabanac’s work on alliesthesia dependent relationship between the need state of the internal body and the perceived pleasure of a stimulus, [88].

Specifically, the relatively low levels of BodyFat in younger SLs may trigger liking for readily available sources of energy, which include sweet-tasting stimuli, whereas the increased FFM in older SLs with its well-established link to increased energy requirements [68] may overrule the negative feedback from the relatively high fat mass and, hence, formulate positive hedonic responses to high levels of sweetness. This would be entirely consistent with early reports that prevalence of the SD phenotype was greater in those who were obese than in those being normal-weight [40,43]. More recently, Coldwell and colleagues proposed a similar relationship between sweet-liking and biological/internal needs in adolescents: those classified into the high sucrose preference phenotype showed stronger signs of active growth, as assessed by a bone-growth biomarker [89]. These findings were later replicated in a cohort of 5–10 year old children [90]. Critically, for hedonic responses to sweetness to represent the internal need state of the body, efficient interoceptive mechanisms need to be in place. Growing evidence suggests an association between Western lifestyle and poor interoceptive abilities [91,92]. While we did not obtain any objective measure of interoception here, in those with shorter exposure to the obesogenic environment (i.e., our younger group), positive hedonic responses to high sweetness (as alliesthesia would dictate) could be interpreted as a reflection of the internal state of the body. Our data may then suggest longer exposure to an obesogenic environment undermines a role for sweetness as an expression of homeostatic state. However, it was notable that the SLs retained relatively high interoceptive abilities, even in the older group. If this holds true, one would expect these presumably intact interoceptive abilities to balance signals from increased FFM against homeostatic mechanisms responding to fat tissue, thereby preventing weight gain.

Our findings suggest a factor that might make SLs less resilient to the temptation of highly liked sweet-tasting foods and drinks is their enhanced reward sensitivity. A conceptual model that distinguishes between the homeostatic and hedonic drives of consumption is the hedonic hunger model; this model posits that desire to eat is expressed in response to seek for pleasure in the absence of physical hunger [93]. Thus, higher sensitivity to reward in our SLs may be explained by an underlying stronger pleasure-seeking trait. Indeed, in the current dataset, while heightened reward sensitivity was found in SLs of both age groups, relatively higher scores were observed in the older group who had been exposed to an obesogenic environment for longer. Further, a positive feedback loop between repeated consumption of palatable foods and hedonic hunger through effects on incentive salience (i.e., desire for a rewarding stimulus) has been suggested [94]. The fact that that sweetened beverages partially explained the relationship between phenotype and anthropometrics among our older participants, but failed to do so in the younger groups, confirms that this might be the case. Essentially, when the obesogenic environment drives food choices, liking for sweetness is likely to be a stronger determinant of dietary intake, and this might be why SLs end up being heavier while SDs are leaner, at least in older individuals. In other words, hedonic responses to sweetness seem to be driven by the relative balance of two factors: need-state and desire for pleasure, as alliesthesia and hedonic hunger would suggest, respectively.

### 4.5. Strength and Limitations

Here, we used a statistically robust method to classify participants into groups of distinct sweet-liking patterns. Furthermore, we collected multiple obesity-related anthropometric measures with sensory and anthropometric measures obtained on separate lab visits to ensure all measures were made under optimal testing conditions, that is, control for extreme hunger or thirst at the time of the taste test and overnight fast and water abstinence for accurate body composition measures. Acknowledging the need for direct between-country contrasts to delineate better the effect of different geographical regions on the drivers of obesity [52], the experimental protocol that was initially designed for the UK was then replicated in a similar population in the US. Some limitations of this study should be noted. Firstly, some consideration should be given to the low statistical power of part of our subgroup analysis (analysis per age group or per country); follow-up studies to confirm those observations are warranted. Secondly, while the two cohorts were matched in proportions of women and men, and we controlled for sex in analyses when appropriate, more women than men were recruited. In terms of ethnicity, Caucasians dominated both cohorts and therefore, our findings may not generalize to other ethnic groups. Additionally, our exploration of dietary correlates was limited to using FFQs for beverages only. Time and funding restrictions meant we were not able to use a detailed dietary intake tool (e.g., dietary recalls, food diaries) in both countries which may have allowed for inclusion of additional food groups or targeted macronutrients in our models. Finally, in the absence of a standardized FFQ suitable for both UK and US populations, our analysis relied on the comparability of different FFQs in the two cohorts. Still, since most of the convincing evidence of sugars’ involvement in obesity derives from research on simple sugars consumed in the form of beverages e.g., in [28], we were able to generate clear and relevant findings from the FFQ data which can be extended in the future by more detailed dietary analysis for the different phenotypes.

## 5. Conclusions

Here, we propose individual variation in liking for sweetness might be a potent candidate towards improved understanding of obesity etiology. To that end, our findings may be of use in adapting tailored health messaging and promotion to each sweet-liking phenotype. That is, developing behavioural techniques to regulate reward sensitivity in SLs and resetting homeostatic eating in SDs. The observed differential effects of sweet-liking patterns on anthropometry that depend on age or more broadly on the level of exposure to the obesogenic environment (i.e., worse anthropometric measures in younger SDs and older SLs) alongside our novel finding that FFM is the body composition compartment most strongly linked to sweet-liking patterns, should be a focus of attention by future studies. To that end, during protocol design and results interpretation, careful consideration of the targeted age group (s) or targeted population (s) in relation to their exposure to the obesogenic environment should be given.

## Figures and Tables

**Table 1:** Adjusted main effects (one-way analysis of covariance) of the sweet-liking phenotype on anthropometrics per age group per country and for the entire sample.

	<21 Years					≥21 Years				
	*F*	*df*	*p*	*ηp^2^*	*n*	*F*	*df*	*p*	*ηp^2^*	*n*
**log_10_ BMI**										
Overall ^1^	0.427	2	0.653	0.007	125	3.040	2	0.051	0.047	125
UK	0.987	2	0.377	0.021	95	0.252	2	0.778	0.012	46
US ^1^	0.326	2	0.725	0.024	30	3.820	2	0.026	0.091	79
**Total Body Fat**										
Overall	3.062	2	0.050	0.048	125	1.521	2	0.233	0.025	125
UK	5.502	2	0.006	0.108	95	0.375	2	0.690	0.018	46
US	0.245	2	0.784	0.019	30	3.557	2	0.033	0.087	79
**Fat Free Mass**										
Overall	0.044	2	0.957	0.001	125	6.524	2	0.002	0.097	125
UK	0.165	2	0.848	0.004	95	2.176	2	0.126	0.094	46
US	0.190	2	0.828	0.014	30	4.679	2	0.012	0.111	79
**Waist Circumference**										
Overall	1.612	2	0.204	0.026	125	3.194	2	0.044	0.050	125
UK	2.309	2	0.105	0.048	95	1.221	2	0.305	0.055	46
US	0.037	2	0.964	0.003	30	4.598	2	0.013	0.109	79
**Waist to Hip Ratio**										
Overall	1.080	2	0.343	0.018	125	2.761	2	0.067	0.044	125
UK	0.717	2	0.491	0.016	95	1.438	2	0.249	0.064	46
US	0.240	2	0.788	0.018	30	2.983	2	0.057	0.074	79

Statistically significant results (*p* < 0.05) and medium or large effect sizes (*ηp*^2^ > 0.06) are bolded. ^1^ F statistics from analysis of variance (no adjustment for sex). BMI, body mass index.

**Table 2:** Food frequency data by phenotype and by country.

		All ^1^	SL	IU		SD			
*Mean (SEM)*									
**Fruit Juice** (glasses/week)									
Overall	1.65 (0.16)		1.64 (0.21)		1.66 (0.27)		1.60 (0.38)	
UK	2.04 (0.28) ^†^		1.68 (0.28)		2.09 (0.45)		2.35 (0.77)	
US	1.20 (0.14)		1.56 (0.33)		1.13 (0.21)		0.96 (0.22)	
**Concentrated Juice Drinks, or Any Juice Drink with Added Sugar** (glasses/week)									
Overall	1.35 (0.24)		1.57 (0.58)		1.25 (0.34)		1.36 (0.37)	
UK	1.25 (0.33)		0.85 (0.19)		1.49 (0.61)		1.37 (0.69)	
US	1.47 (0.36) ^†^		2.75 (1.48)		0.97 (0.18)		1.35 (0.38)	
**Energy/Sports Drinks or Sweetened Caffeinated Drinks** (glasses/week)							
Overall	0.61 (0.06)		0.65 (0.12)		0.51 (0.04)		0.82 (0.20)	
UK	0.53 (0.06)		0.53 (0.10)		0.50 (0.07)		0.64 (0.22)	
US	0.70 (0.11) ^†^		0.84 (0.28)		0.52 (0.06)		0.96 (0.33)	
**Soft Drinks** (glasses/week)							
Overall	1.30 (0.12)		1.07 (0.14)		1.35 (0.19)		1.57 (0.31)	
UK	1.24 (0.17)		0.94 (0.16)		1.39 (0.29)		1.41 (0.40)	
US	1.37 (0.18)		1.29 (0.25)		1.29 (0.25)		1.71 (0.46)	
**Diet Soft Drinks** (glasses/week)							
Overall	1.20 (0.20)		1.42 (0.57)		1.33 (0.25)		0.74 (0.14)	
UK	1.10 (0.16)		1.05 (0.18)		1.26 (0.29)		0.83 (0.23)	
US	1.33 (0.39)		2.02 (1.49)		1.42 (0.42)		0.67 (0.16)	
**Tea or Coffee** (cups/day)							
Overall	1.61 (0.09)		1.35 (0.15)		1.69 (0.14)		1.73 (0.18)	
UK	1.90 (0.13) ^†^		1.63 (0.21)		1.96 (0.20)		2.22 (0.24)	
US	1.26 (0.11)		0.90 (0.17)		1.36 (0.18)		1.32 (0.25)	
**Wine** (glasses/week) ^2^							
Overall	1.98 (0.18)		1.95 (0.31)		1.85 (0.29)		1.89 (0.32)	
UK	1.54 (0.41)		1.52 (0.45)		1.58 (0.74)		1.46 (0.56)	
US	2.21 (0.18) ^†^		2.27 (0.43)		1.99 (0.23)		2.10 (0.39)	
**Beer, Cider, or Cooler** (half pints/week) ^2^							
Overall	2.62 (0.19)		2.52 (0.40)		2.81 (0.25)		2.34 (0.47)	
UK	2.45 (0.33)		3.20 (0.73)		2.20 (0.42)		2.05 (0.66)	
US	2.70 (0.23)		2.03 (0.43)		3.11 (0.31) *		2.49 (0.64)	
**Spirits/Hard Liquor** (shots/week) ^2^							
Overall	1.30 (0.17)		2.00 (0.61)		1.25 (0.17)		0.83 (0.16)	
UK	1.60 (0.43)		3.25 (1.33) *		1.18 (0.37)		0.47 (0.09)	
US	1.14 (0.13)		1.10 (0.35)		1.29 (0.19)		1.01 (0.23)	

The asterisk (*) indicates a significant effect (*p* < 0.05 for one-way ANOVAs) of phenotype on frequency of consumption of the item under investigation within each row. The dagger (†) denotes a cross country significant difference (*p* < 0.05 for Student’s t-tests) in the frequency of consumption of the relevant item under investigation. To facilitate interpretation of the food frequency data, the non-log transformed values are displayed while they are coded as a weekly consumption of a standard portion; habitual intake of tea or coffee is expressed in daily units. ^1^ Data presented refer to the study samples before the clustering process i.e., participants with erratic responses to the sweet taste test are included. ^2^ Data presented are for the subgroup of participants aged 21 or older only. SEM, standard error or the mean.

## Supplementary Materials

The following are available online at https://www.mdpi.com/2072-6643/12/9/2702/s1, Table S1: Behavioural characteristics by country; Table S2: Lifestyle characteristics by country; Table S3: Anthropometric characteristics by country; Table S4: Interaction effects of age on phenotypic differences in anthropometric measures.
