# Supplementary material for: Effects of Sweet-Liking on Body Composition Depend on Age and Lifestyle: A Challenge to the Simple Sweet-Liking—Obesity Hypothesis
Source: Nutrients. 2020 Sep 4;12(9):2702. doi: 10.3390/nu12092702 (PMC7551752; doi:10.3390/nu12092702)
Supplement: Supplementary file 1 [file nutrients-12-02702-s001.pdf]

nutrients

MDPI

Supplementary Material

Table S1. Behavioural characteristics by country.

	UK $N = 146$	US $N = 124$
	Mean (SEM)	
**Three Factor Eating Questionnaire**		
Restraint eating	7.54 (.364)	9.44 (.407)*
Disinhibition	5.59 (.242)	6.31 (.290)
Trait hunger	6.77 (.292)	5.70 (.293)*
**Barratt's Impulsiveness Scale**		
Attentional impulsivity	16.95 (.308)	15.98 (.317)*
Motor impulsivity	22.10 (.370)	21.18 (.345)
Non-planning impulsivity	24.68 (.380)	21.87 (.396)*
**Sensitivity to Punishment –Sensitivity to Reward Questionnaire**		
Sensitivity to Punishment	12.07 (.425)	12.49 (.484)
Sensitivity to Reward	12.36 (.304)	11.46 (351)
**Arnett's Inventory of Sensation Seeking**		
Intensity seeking	24.64 (.353)	23.36 (.385)
Novelty seeking	27.49 (.366)	25.91 (.362)*

The asterisk (*) indicates a significant cross-country difference ($p < .05$) for the values within the same row after adjusting for age. Data from participants (UK: $n = 2$; US: $n = 2$) opting for the answer phrased as 'I am currently on a diet' at post-recruitment are not presented.

Table S2. Lifestyle characteristics by country.

	**UK**<br>*N* = 146	**US**<br>*N* = 124
	**N (%)**	
**Dieting**		
Never	93 (63.7)	71 (57.3)
Once or more times in the past	53 (36.3)	53 (42.7)
**Breakfast consumption***		
5-7 days/week	116 (79.4)	87 (70.2)
2-4 days/week	23 (15.8)	19 (15.3)
0-1 days/week	7 (4.8)	18 (14.5)
**Physical activity level**		
Low	13 (8.9)	8 (6.5)
Moderate	51 (34.9)	35 (28.2)
High	82 (56.2)	81 (65.3)
	Mean (SEM)	
**Average sleep duration** (hours/day)	8.84 (.104)	8.29 (.075)*

SEM, Standard Error of the Mean. All frequencies reported refer to valid percentages. The asterisk (*) indicates a significant cross-country difference ($p < .05$) for the values within the same row or under the same variable after adjusting for age. Data from participants (UK: $n = 2$; US: $n = 2$) opting for the answer phrased as ‘I am currently on a diet’ at post-recruitment are not presented.

Table S3. Anthropometric characteristics by country.

	UK<br>N = 145	US<br>N = 116
	Median (Q1, Q3)	
**BMI** (kg/m^2)	22.1 (20.6, 24.4)	23.6 (21.2, 27.7)
	Mean (SEM)[1]	
**Total body fat** (%)	23.7 (.552)	26.1 (.619)
**Fat free mass** (kg)	49.41 (.475)	50.77 (.532)
**Waist circumference** (cm)	74.9 (.889)	81.5 (.995)*
**Waist circumference to hip circumference ratio**	.767 (.006)	.798 (.006)*

BMI, body mass index; Q1, 25th percentile; Q3, 75th percentile; SEM, Standard Error of the Mean. The asterisk (*) indicates a significant cross-country difference ($p < .05$) for the values within the same row after adjusting for age. [1]All Mean (SEM) values are the estimated marginal means as they were calculated after considering the effects of sex.

Table S4. Interaction effects of age on phenotypic differences in anthropometric measures

	B (SE)	*β*	**95%*CI* *(Lower Bound, Upper Bound)***	*t*	*p*	R^2
**log10BMI**						
Step 1						.004
Constant	1.378 (.013)		(1.353, 1.403)	109.235	.000	
Phenotype	-.006 (.006)	-.060	(-.018, .006)	-.941	.348	
Step 2						.084
Constant	1.004 (.081)		(.8441, .164)	12.344	.000	
Phenotype	-.008 (.006)	-.084	(-.020, .003)	-1.379	.169	
$\log_{10}$Age	**.282 (.061)**	**.284**	**(.162, .401)**	**4.648**	**.000**	
**Total Body Fat**						
Step 1						.377
Constant	16.674 (1.331)		(14.051, 19.296)	12.523	.000	
Phenotype	.137 (.605)	.011	(-1.055, 1.328)	.226	.821	
Sex	11.280 (.923)	.616	(9.461, 13.098	12.218	.000	
Step 2						.410
Constant	-15.671 (8.415)		(-32.246, .905)	-1.862	.064	
Phenotype	-.134 (.592)	-.011	(-1.301, 1.033)	-.226	.821	
Sex	12.090 (.922)	.661	(10.275, 13.906)	13.117	.000	
$\log_{10}$Age	**24.069 (6.188)**	**.195**	**(11.881, 36.257)**	**3.890**	**.000**	
**Fat Free Mass**						
Step 1						.687
Constant	64.653 (1.130)		(62.426, 66.880)	57.190	.000	
Phenotype	-1.150 (.514)	-.080	(-2.161, -.138)	-2.238	.026	
Sex	-17.920 (.784)	-.817	(-19.464, -16.377)	-22.861	.000	
Step 2						.693
Constant	48.277 (7.286)		(33.927, 62.627)	6.626	.000	
Phenotype	-1.287 (.513)	-.089	(-2.297, -.276)	-2.509	.013	
Sex	-17.510 (.798)	-.798	(-19.082, -15.938)	-21.943	.000	
$\log_{10}$Age	**12.186 (5.357)**	**.082**	**(1.635, 22.738)**	**2.275**	**.024**	

**Waist Circumference**						
Step 1						.093
Constant	84.888 (2.190)		(80.575, 89.202)	38.765	.000	
Phenotype	-.999 (.995)	-.061	(-2.958, .961)	-1.004	.316	
Sex	-7.582 (1.518)	-.304	(-10.573, -4.592)	-4.994	.000	
Step 2						.171
Constant	18.948 (13.611)		(-7.861, 45.757)	1.392	.165	
Phenotype	-1.551 (.958)	-.095	(-3.438, .336)	-1.619	.107	
Sex	-5.930 (1.491)	-.238	(-8.866, -2.994)	-3.978	.000	
log_{10}Age	**49.070 (10.008)**	**.292**	**(29.358, 68.782)**	**4.903**	**.000**	
**Waist to Hip Ratio**						
Step 1						.149
Constant	.836 (.014)		(.809, .863)	60.172	.000	
Phenotype	-.007 (.006)	-.066	(-.019, .005)	-1.114	.266	
Sex	-.062 (.010)	-.381	(-.081, -.043)	-6.471	.000	
Step 2						.178
Constant	.562 (.089)		(.387, .737)	6.335	.000	
Phenotype	-.009 (.006)	-.087	(-.022, .003)	-1.493	.137	
Sex	-.055 (.010)	-.339	(-.075, -.036)	-5.708	.000	
log_{10}Age	**.204. (065)**	**.185**	**(.075, .332)**	**3.124**	**.002**	

BMI, Body Mass Index; CI, Confidence Interval; SE, Standard Error

© 2020 by the authors. Submitted for possible open access publication under the terms and conditions of the Creative Commons Attribution (CC BY) license (http://creativecommons.org/licenses/by/4.0/).
